# Supplementary figures and images for: Characteristics of planktonic and sediment bacterial communities in a heavily polluted urban river
Source: PeerJ. 2021 Feb 24;9:e10866. doi: 10.7717/peerj.10866 (PMC7912603; doi:10.7717/peerj.10866)

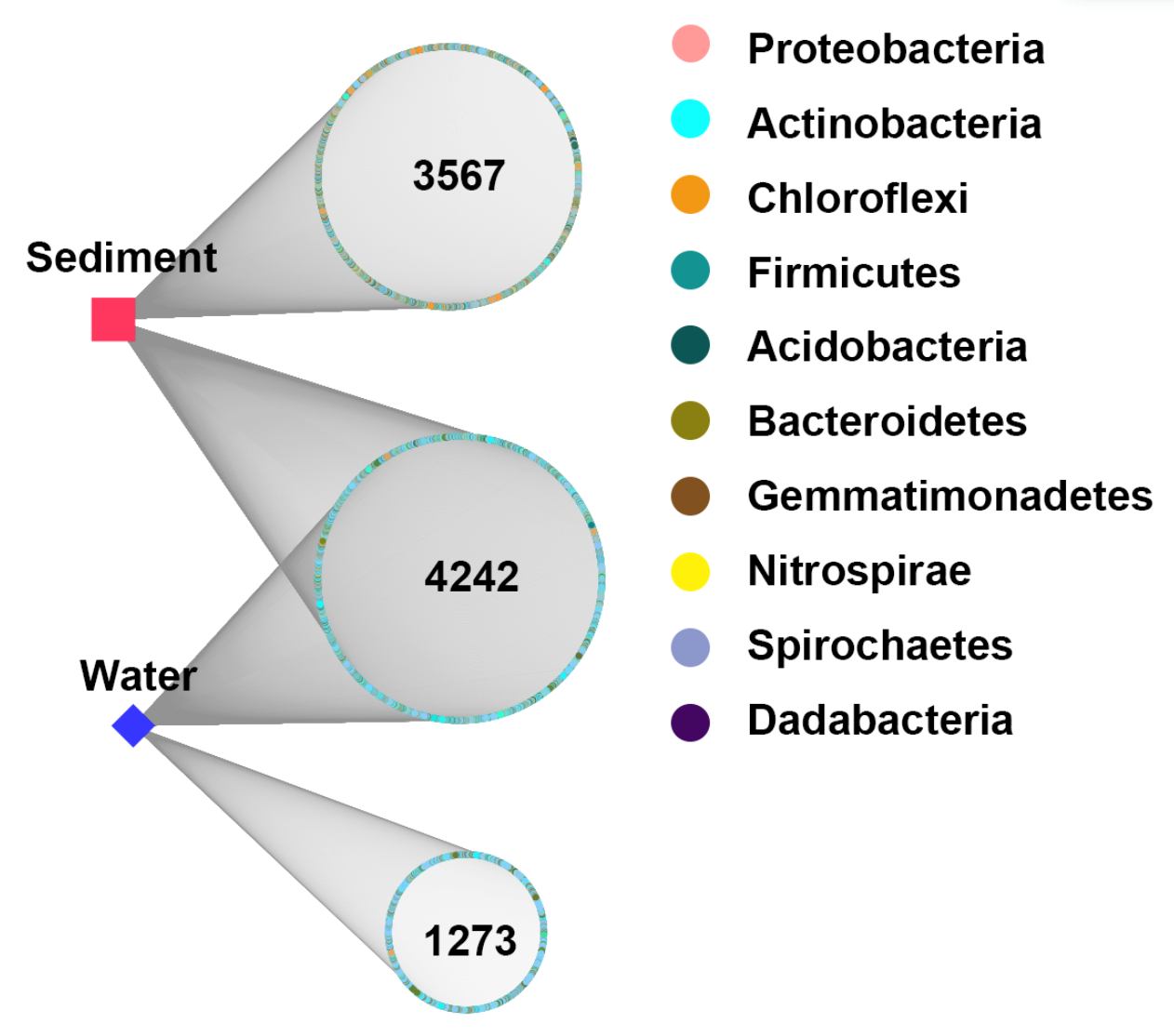

Supplement: Supplemental Information 1 [file peerj-09-10866-s001.png]

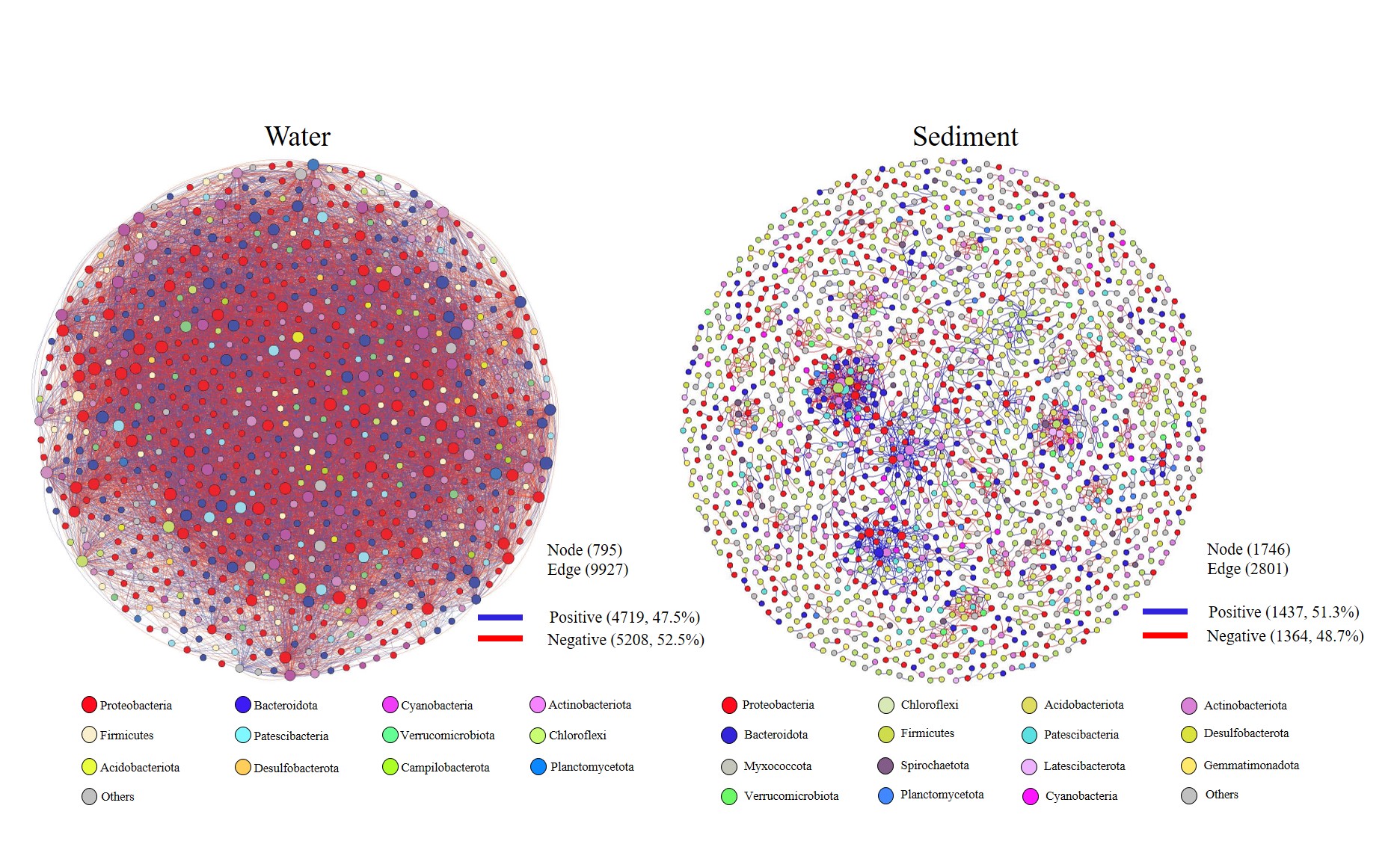

Supplement: Supplemental Information 2 [file peerj-09-10866-s002.jpg]
