# Supplementary material for: Characteristics of planktonic and sediment bacterial communities in a heavily polluted urban river
Source: PeerJ. 2021 Feb 24;9:e10866. doi: 10.7717/peerj.10866 (PMC7912603; doi:10.7717/peerj.10866)
Supplement: Supplemental Information 3 [file peerj-09-10866-s003.docx]

**Table S1** Richness and diversity of the bacterial libraries in the different sediment and water samples. Samples SFe1, SFe2, SFe3, SFe4 and SFe5 represent the February sediment samples from sites L1–L5, respectively. Samples SAu1, SAu2, SAu3, SAu4 and SAu5 represent the August sediment samples from sites L1–L5, respectively. Samples WFe1, WFe2, WFe3, WFe4 and WFe5 represent the February water samples from sites L1–L5, respectively. Samples WAu1, WAu2, WAu3, WAu4 and WAu5 represent the August water samples from sites L1–L5, respectively.

| Sample | Reads | OTUs | Chao1 estimator | Shannon index | Simpson index | Coverage(%) |
| --- | --- | --- | --- | --- | --- | --- |
| WFe1 | 55229 | 1706 | 3159 | 4.91 | 0.0267 | 97.2 |
| WFe2 | 60319 | 1760 | 2865 | 4.95 | 0.0324 | 97.8 |
| WFe3 | 49933 | 1757 | 2954 | 4.84 | 0.0302 | 97.7 |
| WFe4 | 56449 | 2011 | 3424 | 5.04 | 0.0229 | 97.4 |
| WFe5 | 63295 | 1860 | 3110 | 4.91 | 0.0256 | 98.1 |
| WAu1 | 30221 | 2130 | 3602 | 4.49 | 0.0449 | 98.3 |
| WAu2 | 34772 | 2434 | 3810 | 4.72 | 0.0411 | 98.3 |
| WAu3 | 35718 | 2106 | 3337 | 4.83 | 0.0282 | 98.2 |
| WAu4 | 36284 | 2626 | 3833 | 5.18 | 0.0208 | 98.1 |
| WAu5 | 43954 | 2783 | 3785 | 5.20 | 0.0214 | 98.4 |
| SFe1 | 45759 | 3488 | 4968 | 7.05 | 0.0021 | 95.5 |
| SFe2 | 57666 | 3851 | 5338 | 6.83 | 0.0045 | 96.0 |
| SFe3 | 55226 | 3645 | 5064 | 6.88 | 0.0030 | 95.9 |
| SFe4 | 51440 | 3490 | 4806 | 6.69 | 0.0049 | 96.1 |
| SFe5 | 55582 | 3892 | 5270 | 7.02 | 0.0024 | 96.3 |
| SAu1 | 28133 | 3766 | 4515 | 7.09 | 0.0020 | 97.9 |
| SAu2 | 34726 | 4731 | 6015 | 7.06 | 0.0034 | 97.6 |
| SAu3 | 32575 | 4109 | 5324 | 6.73 | 0.0050 | 97.7 |
| SAu4 | 32899 | 3235 | 4083 | 6.47 | 0.0077 | 98.3 |
| SAu5 | 36699 | 3847 | 5129 | 6.40 | 0.0090 | 97.8 |

**Table S2** The topological property of microbial network between sediment and water based on the OTU based on the sequencing data.

| topological property | Sediment | Water |
| --- | --- | --- |
| total nodes | 1746 | 795 |
| total edges | 2801 | 9927 |
| negative edges | 1364 | 5208 |
| positive edges | 1437 | 4719 |
| Average connectivity | 3.208 | 24.974 |
| Average clustering coefficient (avgCC) | 0.095 | 0.215 |
| Average length | 10.152 | 4.322 |
| R square of power-law | 0.902 | 0.477 |
| module | 235 | 98 |
| modularity | 0.922 | 0.161 |

**Table S3** Percent of potential bacterial pathogen sequences at the genus level in HS and RI areas.

| Phyla | Genus | Sediment | | | | Water | | | |
| --- | --- | --- | --- | --- | --- | --- | --- | --- | --- |
|  |  | Winter | | Summer | | Winter | | Summer | |
|  |  | HS | RI | HS | RI | HS | RI | HS | RI |
| *Proteobacteria* | *Pseudomonas* | 0.148 | 0.218 | 0.036 | 0.072 | 0.395 | 0.488 | 0.929 | 2.876 |
|  | *Arcobacter* | 0.006 | 0.010 | 0.000 | 0.002 | 1.679 | 3.954 | 0.928 | 4.877 |
|  | *Acinetobacter* | 0.024 | 0.018 | 0.002 | 0.004 | 0.109 | 0.173 | 0.887 | 1.390 |
|  | *Aeromonas* | 0.010 | 0.005 | 0.001 | 0.000 | 0.038 | 0.044 | 0.420 | 0.822 |
|  | *Legionella* | 0.015 | 0.036 | 0.011 | 0.013 | 0.027 | 0.024 | 0.053 | 0.092 |
|  | *Escherichia-Shigella* | 0.205 | 0.924 | 0.008 | 0.337 | 0.302 | 0.513 | 0.049 | 0.131 |
|  | *Enterobacter* | 0.010 | 0.052 | 0.000 | 0.027 | 0.020 | 0.033 | 0.136 | 0.310 |
| *Bacteroidetes* | *Bacteroides* | 0.063 | 0.027 | 0.006 | 0.003 | 0.287 | 0.749 | 0.144 | 0.855 |
|  | *Flavobacterium* | 0.323 | 0.453 | 0.050 | 0.160 | 0.000 | 0.000 | 3.323 | 4.490 |
| *Actinobacteria* | *Mycobacterium* | 0.619 | 0.473 | 0.290 | 0.434 | 1.118 | 2.114 | 0.459 | 0.552 |
| *Firmicutes* | *Bacillus* | 0.123 | 0.624 | 0.446 | 0.899 | 0.045 | 0.078 | 0.156 | 0.294 |
|  | *Clostridium* | 0.771 | 1.300 | 0.680 | 1.667 | 0.163 | 0.119 | 0.169 | 0.152 |
| Total |  | 2.316 | 4.140 | 1.531 | 3.618 | 4.182 | 8.289 | 7.653 | 16.841 |

**Table S4** The physicochemical parameters of each water sample. Samples WFe1, WFe2, WFe3, WFe4 and WFe5 represent the February water samples from sites L1–L5, respectively. Samples WAu1, WAu2, WAu3, WAu4 and WAu5 represent the August water samples from sites L1–L5, respectively.

| Sample | Longitude & Latitude | pH | Temperature | NH_4_^+^-N | NO_3_^-^-N | TN | TP | TOC |
| --- | --- | --- | --- | --- | --- | --- | --- | --- |
|  |  |  | (℃) | (mg/L) | (mg/L) | (mg/L) | (mg/L) | (mg/L) |
| WFe1 | 106°24′43.81″ | 7.81±0.12 | 14.5±0.2 | 1.37±0.09 | 3.46±0.31 | 9.55±0.39 | 0.83±0.03 | 42.85±0.80 |
|  | 29°48′13.38″ |  |  |  |  |  |  |  |
| WFe2 | 106°21′29.20″ | 7.77±0.08 | 13.9±0.2 | 1.86±0.05 | 4.20±0.28 | 10.73±0.43 | 0.93±0.01 | 50.30±0.76 |
|  | 29°43′34.39″ |  |  |  |  |  |  |  |
| WFe3 | 106°21′16.24″ | 7.80±0.03 | 13.8±0.1 | 1.72±0.03 | 1.98±0.20 | 7.08±0.40 | 0.65±0.02 | 45.13±0.55 |
|  | 29°38′26.32″ |  |  |  |  |  |  |  |
| WFe4 | 106°22′34.61″ | 7.74±0.10 | 14.3±0.1 | 1.03±0.11 | 2.80±0.19 | 8.07±0.29 | 0.62±0.02 | 48.52±0.49 |
|  | 29°33′46.60″ |  |  |  |  |  |  |  |
| WFe5 | 106°21′52.05″ | 7.81±0.30 | 14.1±0.2 | 1.45±0.12 | 2.22±0.29 | 4.43±0.36 | 0.53±0.06 | 40.91±0.37 |
|  | 29°30′26.73″ |  |  |  |  |  |  |  |
| WAu1 | 106°24′43.81″ | 7.57±0.21 | 26.8±0.3 | 1.86±0.09 | 2.03±0.10 | 6.42±0.50 | 0.49±0.01 | 56.35±0.98 |
|  | 29°48′13.38″ |  |  |  |  |  |  |  |
| WAu2 | 106°21′29.20″ | 7.82±0.09 | 26.3±0.1 | 2.11±0.19 | 3.07±0.18 | 8.13±0.37 | 0.24±0.01 | 54.13±1.20 |
|  | 29°43′34.39″ |  |  |  |  |  |  |  |
| WAu3 | 106°21′16.24″ | 7.36±0.08 | 27.1±0.2 | 1.77±0.04 | 2.36±0.32 | 4.75±0.30 | 0.38±0.04 | 45.59±0.85 |
|  | 29°38′26.32″ |  |  |  |  |  |  |  |
| WAu4 | 106°22′34.61″ | 7.69±0.13 | 26.3±0.3 | 1.06±0.05 | 3.88±0.17 | 5.08±0.29 | 0.44±0.09 | 35.10±0.45 |
|  | 29°33′46.60″ |  |  |  |  |  |  |  |
| WAu5 | 106°21′52.05″ | 7.92±0.07 | 26.3±0.2 | 1.15±0.10 | 2.11±0.15 | 6.82±0.38 | 0.50±0.06 | 34.69±1.03 |
|  | 29°30′26.73″ |  |  |  |  |  |  |  |

**Table S5** The physicochemical parameters of each sediment sample. Samples SFe1, SFe2, SFe3, SFe4 and SFe5 represent the February sediment samples from sites L1–L5, respectively. Samples SAu1, SAu2, SAu3, SAu4 and SAu5 represent the August sediment samples from sites L1–L5, respectively.

| Sample | Longitude & Latitude | pH | Temperature | NH_4_^+^-N | NO_3_^-^-N | TN | TP | TOC |
| --- | --- | --- | --- | --- | --- | --- | --- | --- |
|  |  |  | (℃) | (mg/kg) | (mg/kg) | (mg/kg) | (mg/kg) | (mg/kg) |
| SFe1 | 106°24′43.81″ | 7.37±0.05 | 14.6±0.2 | 19.22±0.34 | 87.21±0.59 | 0.69±0.02 | 0.64±0.01 | 23.69±0.58 |
|  | 29°48′13.38″ |  |  |  |  |  |  |  |
| SFe2 | 106°21′29.20″ | 7.21±0.03 | 14.5±0.1 | 18.46±0.20 | 83.41±0.91 | 0.59±0.01 | 0.70±0.04 | 35.53±0.61 |
|  | 29°43′34.39″ |  |  |  |  |  |  |  |
| SFe3 | 106°21′16.24″ | 7.08±0.08 | 13.7±0.1 | 15.67±0.29 | 61.77±0.46 | 0.39±0.01 | 0.50±0.01 | 16.33±0.39 |
|  | 29°38′26.32″ |  |  |  |  |  |  |  |
| SFe4 | 106°22′34.61″ | 7.19±0.07 | 14.5±0.1 | 17.34±0.33 | 65.62±0.32 | 0.61±0.05 | 0.87±0.06 | 27.38±0.44 |
|  | 29°33′46.60″ |  |  |  |  |  |  |  |
| SFe5 | 106°21′52.05″ | 7.54±0.11 | 14.4±0.1 | 19.12±0.19 | 66.85±0.67 | 0.58±0.03 | 0.68±0.07 | 24.50±0.29 |
|  | 29°30′26.73″ |  |  |  |  |  |  |  |
| SAu1 | 106°24′43.81″ | 7.36±0.02 | 25.9±0.2 | 18.85±0.21 | 68.39±0.60 | 0.69±0.02 | 0.29±0.01 | 11.37±0.80 |
|  | 29°48′13.38″ |  |  |  |  |  |  |  |
| SAu2 | 106°21′29.20″ | 7.29±0.04 | 26.0±0.3 | 16.29±0.30 | 80.65±0.73 | 0.62±0.07 | 0.38±0.01 | 14.51±0.69 |
|  | 29°43′34.39″ |  |  |  |  |  |  |  |
| SAu3 | 106°21′16.24″ | 7.11±0.13 | 25.9±0.1 | 14.64±0.27 | 53.83±0.55 | 0.35±0.01 | 0.22±0.02 | 8.99±0.43 |
|  | 29°38′26.32″ |  |  |  |  |  |  |  |
| SAu4 | 106°22′34.61″ | 7.33±0.09 | 25.8±0.2 | 13.48±0.19 | 44.97±0.50 | 0.34±0.01 | 0.47±0.04 | 20.36±0.80 |
|  | 29°33′46.60″ |  |  |  |  |  |  |  |
| SAu5 | 106°21′52.05″ | 7.28±0.06 | 25.7±0.1 | 13.97±0.28 | 45.93±0.62 | 0.36±0.02 | 0.32±0.02 | 11.64±0.19 |
|  | 29°30′26.73″ |  |  |  |  |  |  |  |
